# Proteome‐wide assessment of human interactome as a source of capturing domain–motif and domain‐domain interactions

**DOI:** 10.1002/ccs3.12014

**Published:** 2024-01-19

**Authors:** Sobia Idrees, Keshav Raj Paudel

**Affiliations:** ^1^ School of Biotechnology and Biomolecular Sciences University of New South Wales Sydney New South Wales Australia; ^2^ Centre for Inflammation Centenary Institute and the University of Technology Sydney School of Life Sciences Faculty of Science Sydney New South Wales Australia

**Keywords:** DDIs, DMIs, high‐throughput methods, PPIs, proteome, SLiMs

## Abstract

Protein–protein interactions (PPIs) play a crucial role in various biological processes by establishing domain–motif (DMI) and domain–domain interactions (DDIs). While the existence of real DMIs/DDIs is generally assumed, it is rarely tested; therefore, this study extensively compared high‐throughput methods and public PPI repositories as sources for DMI and DDI prediction based on the assumption that the human interactome provides sufficient data for the reliable identification of DMIs and DDIs. Different datasets from leading high‐throughput methods (Yeast two‐hybrid [Y2H], Affinity Purification coupled Mass Spectrometry [AP‐MS], and Co‐fractionation‐coupled Mass Spectrometry) were assessed for their ability to capture DMIs and DDIs using known DMI/DDI information. High‐throughput methods were not notably worse than PPI databases and, in some cases, appeared better. In conclusion, all PPI datasets demonstrated significant enrichment in DMIs and DDIs (*p*‐value <0.001), establishing Y2H and AP‐MS as reliable methods for predicting these interactions. This study provides valuable insights for biologists in selecting appropriate methods for predicting DMIs, ultimately aiding in SLiM discovery.

## Graphical abstract

Protein‐protein interaction datasets generated by high‐throughput methods in human interactome revealed significant enrichment in terms of capturing domain‐motif (DMI) and domain ‐domain interactions offering valuable insights for biologists in selecting appropriate methods for DMI prediction and aiding in Short Linear Motif discovery.

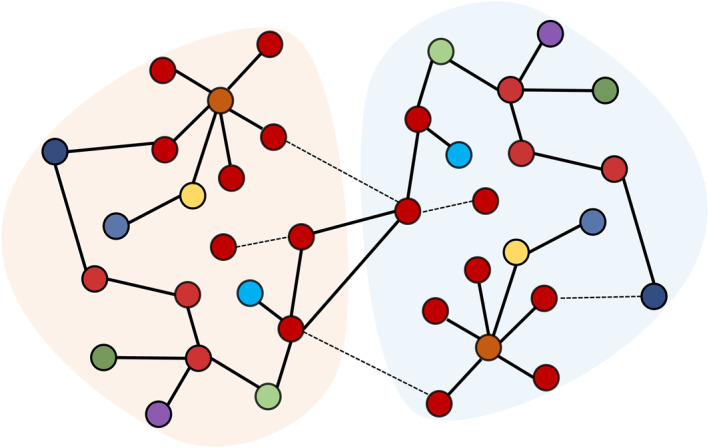

## 1 INTRODUCTION

Protein–protein interactions (PPIs) are indispensable for sustaining essential molecular functions in living cells. They serve various functions, such as catalyzing metabolic reactions, transporting molecules, modifying enzymatic kinetics, and adjusting protein specificity.^1^, ^2^, ^3^ Over the last decade, numerous studies have uncovered PPIs in diverse organisms.^4^, ^5^ The insights gained from these investigations are widely applied to understand the cellular organization of organisms and address diseases, including bacterial/viral infections and cancer, through disruption of signaling events by targeting PPIs.^6^, ^7^ Despite these advancements, the human PPI interactome remains incomplete compared to transcriptome and genome reference sequences.^8^

Predominantly, known PPIs are mediated by two types of modules: domains and short linear motifs (SLiMs). Domains exhibit globular structures formed by long peptides, while SLiMs are linear, recurring functional peptides with 2–15 contiguous residues.^9^, ^10^, ^11^, ^12^, ^13^, ^14^ SLiMs participate in various cellular processes,^15^, ^16^ such as sub‐cellular localization, post‐translational modification (PTM), regulatory functions, protein trafficking, cell cycle control, signal transduction, and stabilization of scaffolding processes.^17^, ^18^ SLiMs are often found in intrinsically disordered protein regions (IDRs)^19^, ^20^, ^21^, ^22^ and interact with domains of other proteins to establish transient and low‐affinity domain–motif interactions (DMIs) in the 1–150 μM range.^22^, ^23^, ^24^ Despite the critical role of DMIs in mediating essential cellular functions, only a fraction has been identified and cataloged in resources such as Eukaryotic Linear Motif (ELM)^23^ and 3DID,^25^ raising concerns that numerous DMIs are yet to be discovered.^2^, ^7^, ^10^, ^12^

Various experimental techniques have emerged in recent years for detecting PPIs, each with advantages and drawbacks. Small‐scale experiments are good at uncovering a limited number of high‐quality PPIs. In contrast, high‐throughput methods can identify numerous PPIs, but the quality of these interactions tends to be lower.^4^, ^5^ Yeast two‐hybrid (Y2H), Affinity Purification coupled Mass Spectrometry (AP‐MS) and Co‐fractionation‐coupled Mass Spectrometry (CoFrac‐MS) are three widely recognized methods that have detected a significant proportion of PPIs.^7^ The data generated by these PPI detection methods are instrumental in studying, protein complexes, biological pathways, and identifying potential drug targets. However, ensuring the biological significance of the knowledge gained from studying PPIs requires focusing on the quality of the detected interactions.^8^, ^26^ High‐throughput methods may inadvertently capture false positive interactions, necessitating experimental and computational validation approaches.^8^, ^26^ Detecting PPIs also faces challenges related to the physiological settings during experiments, as specific conditions influence certain PPIs. Factors such as the PTMs, transient nature of interactions, IDRs and protein abundance can impact PPI detection. Consequently, uncovering a proteome‐wide interactome proves to be a formidable task. To assess the reliability of PPIs, two common approaches are employed: designing new experimental methods for validation or developing computational methods to evaluate reliability by filtering out potential false positives. This involves determining the probability of the observed PPIs.^8^, ^27^

The significance of data generated by current high‐throughput screens in uncovering novel SLiMs and DMIs is acknowledged. However, applying high stringency filtering poses a risk of losing low‐affinity DMIs.^8^, ^26^ Only 1% of DMIs have been identified from high‐throughput data, raising concerns about the potential depletion of DMIs through current methods. It becomes crucial to employ suitable PPI detection techniques when exploring SLiMs or DMIs.^28^ Despite the growing importance, there hasn’t been a dedicated study to validate the effectiveness of current methods in terms of capturing DMIs and DDIs.^3^, ^7^ Therefore, we conducted a comprehensive interactome‐wide comparative study to assess various high‐throughput methods and databases as a source of capturing DMIs and DDIs, which will eventually assist system biologists in selecting appropriate methods for discovering SLiMs or DMIs.

## 2 METHODS

### 2.1 Data collection and pre‐processing

The data on SLiMs was obtained from the ELM database,^23^ renowned for its experimentally validated and manually curated SLiM information. This makes a reliable source for known SLiM mediated interactions. A total of 327 ELM classes (distinct SLiMs), encompassing experimentally validated motif instances (2278 specific protein occurrences), associated interacting domain data (200 ELM interacting domains), and known human DMIs (1236 human DMIs) were retrieved from [http://www.elm.eu.org/] on 2023‐06‐23. Five high‐throughput interaction datasets—HI‐II‐14,^29^ CoFrac‐12,^30^ CoFrac‐15,^31^ BioPlex2.0,^32^ and QUBIC‐15^33^ were retrieved. Additionally, five prominent PPI databases were assessed: BioGrid v4.42,^34^ IntAct,^35^ the High‐quality INTeractome (HINT) database,^36^ the Human Integrated Protein–Protein Interaction rEference (HIPPIE)^38^ and the Human Protein Reference Database (HPRD).^37^ BioGrid and IntAct were narrowed down to human interactions, with datasets mapped onto Uniprot IDs, restricted to pairs of reviewed Uniprot proteins, and treated as nonredundant symmetrical interactions. PPI subsets by experiment type (AP‐MS, Y2H, and Co‐fractionation) were created for BioGrid, IntAct, and HIPPIE. Keywords such as “Two‐hybrid,” “Co‐fractionation,” and “Affinity Capture‐MS” were employed for pulling interactions from the BioGrid database. Only high‐throughput two‐hybrid interactions were selected. For the IntAct database, molecular interaction ontologies (MI:0676, MI:0400, MI:0004, and MI:0018) were utilized to extract specific subsets. The HIPPIE database interactions were obtained using keywords “Affinity‐Capture,” “Two‐hybrid,” and “Co‐fractionation.” All PPI datasets were constrained to reviewed Uniprot protein pairs, made symmetrical, and redundant entries were eliminated. The analysis focused on directed networks featuring specific motif and domain proteins, necessitating the inclusion of both A–B and B–A interactions. The percentage (%) of explained PPIs was calculated using nonredundant symmetrical PPI pairs (i.e., A‐B and B‐A). Additionally, a False Discovery Rate (FDR) for individual DMIs was estimated as the proportion of predicted DMIs explained, on average, by random associations, using the mean random DMI count.

### 2.2 DMI enrichment

To assess enrichment differences in various high‐throughput methods, we utilized known DMI and SLiM information from the ELM database. The analysis was conducted using our previously developed tool, SLiMEnrich v1.5.1,^39^ with iso‐filter, to evaluate enrichment in different PPI datasets. Enrichment estimation employed a permutation test, where proteins were randomly selected to create new interaction pairs without replacement from the original PPI data, ensuring an identical degree for each protein due to permutation without replacement. The datasets underwent 1000 permutations to obtain a robust estimation of random DMIs. Enrichment was quantified as an empirical *p*‐value, corresponding to the probability of observing at least as many DMIs in random PPI data. The DMI enrichment (E‐score) was calculated as the ratio of predicted DMI to the mean (*μ*) random DMI as follows.

$$\text{Escore}=\frac{{\text{DMI}}_{\text{pred}}}{{\mu \text{DMI}}_{\text{rand}}}$$

The total proportion of potential DMIs found in PPIs was determined, representing the proportion of theoretically identifiable DMIs given the proteins in the PPI datasets. The distribution of the real DMI count over 1000 randomizations was estimated as follows:

$${\text{DMI}}_{\text{real}}=O_{\text{obs}}-R_{\text{rand}}$$

${\text{DMI}}_{\text{real}}$ represents the estimated real number of DMIs in the PPI dataset.

$O_{\text{obs}}$ is the number of observed DMIs in the real PPI dataset.

$R_{\text{rand}}$ is the distribution of observed DMIs in the random PPI datasets.

Normalization was performed by dividing the number (*n*) of real DMIs by the mean (*μ*) random DMIs, which is expressed as:

$${\text{DMI}}_{\text{norm}}=\frac{{n\text{DMI}}_{\text{real}}}{{\mu \text{DMI}}_{\text{rand}}}$$

For this analysis, the ELMi‐Protein strategy of SLiMEnrich was applied to assess enrichment in various publicly available datasets. The ELMi‐Protein strategy maps PPI protein pairs directly onto known DMI data in ELM. Additionally, the impact of each ELM type on enrichment was examined to identify whether specific ELM types influenced DMI enrichment differently in various high‐throughput methods. Significance in the capturing of different interactions among datasets was evaluated using Pearson's pairwise chi‐square test. This involved pairwise comparisons of all possible combinations of datasets, with *p*‐values calculated to determine statistical significance.

### 2.3 Assessing DMI prediction quality

To assess the accuracy of DMI predictions, we adopted various strategies. Initially, we utilized the ELMc‐Protein strategy, which gauges enrichment based on known ELM instances provided by the ELM database. To introduce a more realistic noise factor into the DMI network, we incorporated domain information into the equation. This was achieved through the ELMc‐Domain strategy, wherein known ELM instances were aligned with their corresponding Pfam domain partners. The evaluation of DMI enrichment encompassed calculations by SLiMEnrich v1.5.1 and an assessment of the actual DMIs captured from all PPI datasets.

### 2.4 DDI enrichment

We also assessed DDI enrichment by incorporating experimentally validated DDI data from the 3DID database^25^ (https://3did.irbbarcelona.org/download.php). 3DID stands out as a reliable resource for known interactions due to its compilation of high‐resolution 3D structures of established PPIs.^25^ We retrieved PDB Ids of 3D DDI complexes and their interacting chain information from the 3DID database, totaling 15,717 DDIs [retrieved: 2023‐02‐10]. Subsequently, we mapped the interacting PDB chains to their corresponding Uniprot proteins, utilizing the PDBSWS tool.^40^ The resulting DDI protein pairs, comprising 5589 DDIs, were rendered nonredundant and restricted to reviewed Uniprot proteins. This dataset was then employed as a known DDI reference to assess enrichment across different datasets using SLiMEnrich v1.5.1, applying the same methodology as previously defined for DMIs.^39^

### 2.5 Evaluating the impact of PPI prediction quality on DMI enrichment

To gauge the impact of PPI quality, we employed an enrichment‐based evaluation approach. The HIPPIE PPI dataset was stratified into 10 distinct subsets based on their confidence scores, ranging from 0.1 to 1. Each subset was defined as follows: Subset 0.1 encompassed PPIs with confidence scores ranging from 0.11 to 0.19, while subset 0.2 included PPIs with confidence scores ranging from 0.20 to 0.29. Similarly, subset 0.3 consisted of PPIs with confidence scores ranging from 0.31 to 0.39, and so forth. The pattern continued with each subsequent subset. The last subset, subset 1, comprised PPIs exclusively with a confidence score of 1. Then the relationship between PPI confidence score and DMI enrichment was observed for generated groups. Intriguingly, this analysis helped to assess the relationship between these two factors and uncovered whether the quality of PPIs can influence DMI enrichment.

## 3 RESULTS

### 3.1 High‐throughput screens and public PPI resources capture DMIs and DDIs

**FIGURE 1:**
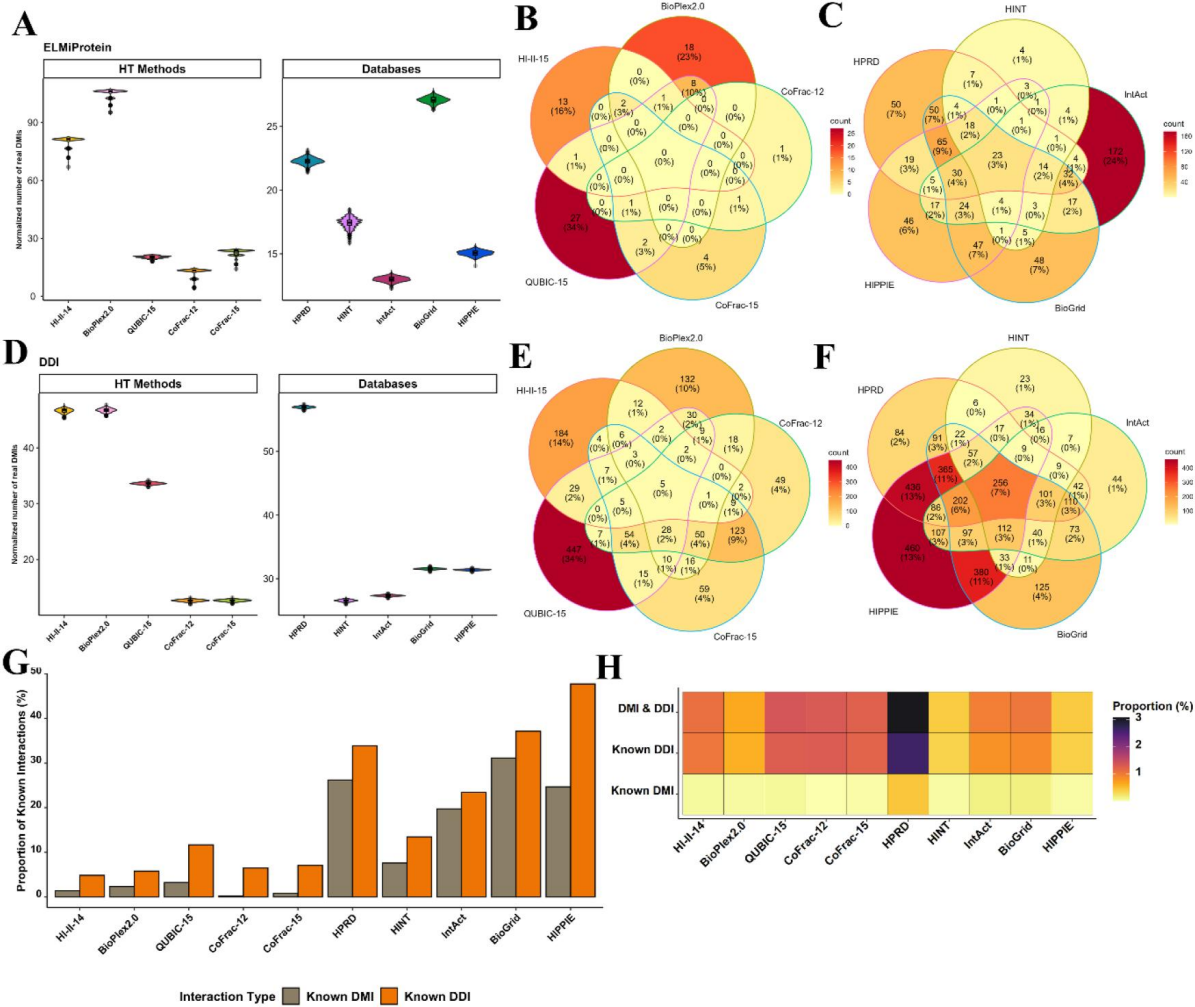
Identification of known DMIs and DDIs from different datasets. (A) Normalized number of DMIs captured over 1000 randomizations. The *Y*‐axis represents the normalized number of DMIs, with each bar depicting the real DMIs captured over 1000 randomizations, calculated by subtracting random DMIs from observed DMIs. (B) Overlap of captured known DMIs between high‐throughput datasets. (C) Overlap of captured known DMIs between PPI databases. (D) Normalized number of DDIs captured over 1000 randomizations. The *Y*‐axis signifies the normalized number of DDIs, and each bar indicates the real DDIs captured over 1000 randomizations, obtained by subtracting random DDIs from observed DDIs. (E) Overlap of captured known DDIs between high‐throughput datasets. (F) Overlap of captured known DDIs between PPI databases. (G) Total proportion of DMIs and DDIs captured from the known human DMIs (1236 DMIs) and known human DDIs dataset (5589 DDIs). (H) Percentage of PPIs that are known DMIs, DDIs, or both (nonredundant and reviewed proteins only). The *Y*‐axis depicts the percentage of PPIs that can be explained as DMIs or DDIs. DDI, domain–domain interaction; DMI, Domain Motif Interactions; PPI, Protein–protein interaction.

This research involves a comparison of five distinct proteome‐wide human interactomes and five publicly accessible databases. The aim is to assess their effectiveness in capturing DMIs and DDIs. PPIs were made nonredundant (NR) and symmetrical, and analysis was restricted to reviewed UniProt protein pairs of the human interactome. The NR PPI pairs were used to determine domain motif enrichment using SLiMEnrich v1.5.1.^39^ Each dataset demonstrated notable enrichment (*p*‐value <0.05), indicating their proficiency in capturing DMIs. The BioPlex2.0 AP‐MS dataset consisted of 53,710 symmetrical and nonredundant PPIs, with merely 29 belonging to the known DMIs in the ELM database. Despite initial impressions, permutation testing uncovered an enrichment of around 106 times the anticipated number of known DMIs that would result from random association of the 53,710 PPIs (*p* < 0.001). Conversely, the second AP‐MS dataset, QUBIC‐15, captured 40 known DMIs in 50,573 PPIs, exhibiting comparatively lower enrichment compared to BioPlex2.0. HI‐II‐14, where PPIs were predicted using the Y2H method, had only 17 known DMIs. Enrichment for HI‐II‐14 was also high (81×) showing that the Y2H screen also captures DMIs. Co‐fractionation methods, however, captured only a few known DMIs (CoFrac‐12 = 3 DMIs, CoFrac‐15 = 10 DMIs) (Table 1, Figure 1A). BioPlex2.0 and QUBIC‐15 shared a 10% overlap between identified DMIs, while the rest of the methods only shared a small fraction of 1%–3% in identified DMIs (Figure 1B). To further evaluate the effectiveness of PPI data in capturing DMIs, we expanded our analysis to include various comprehensive PPI databases featuring information from high‐throughput studies. Specifically, we selected five prominent databases—IntAct, BioGrid, HPRD, HINT, and HIPPIE—to predict DMIs within the human interactome. BioGrid ranked highest in terms of enrichment, followed by HPRD and HINT. While HIPPIE captured more DMIs than other databases, it exhibited the lowest level of enrichment. Moreover, the enrichment scores of all other datasets were lower than those observed in HI‐II‐14 and BioPlex2.0 (Table 1, Figure 1A). The overlap between the known DMIs between all databases was quite low (1%–4%) (Figure 1C).

**TABLE 1:** Assessment of different PPI resources in terms of capturing DMIs and DDIs.

Dataset	PPIs^a^	Method	potDMIs^b^	DMIs^c^	DMI enrichment^d^ (4 s.f)	potDDIs^e^	DDIs^f^	DDI enrichment^g^ (4 s.f.)
HI‐II‐14^29^	25,956	Y2H	119	17	81.34**	1272	271	47.65**
[retrieved: 2023‐06‐01]								
BioPlex2.0^32^	53,710	AP‐MS	226	29	106.2**	2295	324	47.75**
[retrieved: 2023‐06‐01]								
QUBIC‐15^33^	50,573	AP‐MS	292	40	21.27*	2149	653	34.63**
[retrieved: 2023‐06‐01]								
CoFrac‐12^30^	27,643	CoFrac‐MS	104	3	13.39**	1544	362	13.64**
[retrieved: 2023‐06‐01]								
CoFrac‐15^31^	32,452	CoFrac‐MS	144	10	23.64**	1810	395	13.65**
[retrieved: 2023‐06‐01]								
HPRD^37^	71,811	All	773	324	23.27**	4234	1893	57.94**
[retrieved: 2023‐06‐01]								
HINT^36^	203,733	All	765	94	18.43**	4227	753	27.57**
[retrieved: 2023‐06‐01]								
IntAct^35^	159,377	All	880	244	14**	4643	1311	28.24**
[retrieved: 2023‐04‐23]								
BioGrid v4.4.2^34^	235,458	All	842	385	28.07**	4633	2075	32.54**
[retrieved: 2023‐06‐01]								
HIPPIE v2.1^38^	689,858	All	890	305	16.06**	5000	2667	32.39**
[retrieved: 2023‐06‐01]								

Abbreviations: CoFrac‐MS, Co‐Fraction‐coupled Mass Spectrometry; DDI, domain–domain interaction; DMI, Domain Motif Interactions; HINT, High‐quality Interactome; HPRD, Human Protein Reference Database; PPI, Protein–protein interaction; SLiM, Short Linear Motifs.

^a^ Count of symmetrical and nonredundant PPIs involving Uniprot‐reviewed protein pairs.

^b^ Total count of all possible DMIs based on the proteins present in each dataset.

^c^ Known interactions between SLiMs and proteins sourced from the ELM database, presented as a percentage captured from potential DMIs.

^d^ Actual enrichment of known DMIs captured from PPIs.

^e^ Total count of all possible DDIs based on the proteins in each dataset.

^f^ Known interactions between domain–domain pairs from the 3DID database, expressed as a percentage captured from potential DDIs.

^g^ Actual enrichment of known DDIs captured from PPIs.

*p‐value <0.05, **p‐value <0.001.

We also assessed the effectiveness of these datasets in capturing DDIs. To achieve this, we utilized established DDI data from the 3DID database^25^ and examined the enrichment across different datasets. Among high‐throughput screens, BioPlex2.0 demonstrated the highest enrichment, with HI‐II‐14 and QUBIC‐15 following suit. The CoFrac datasets also exhibited noteworthy enrichment in terms of DDIs (Figure 1D). However, only a minor fraction of DDIs was shared among all methods (Figure 1E). Among databases, HPRD emerged as the most enriched dataset for capturing DDIs, trailed by BioGrid, HIPPIE, IntAct, and HINT (Table 1, Figure 1B). Interestingly, all databases had 7% of known DDIs in common across them (Figure 1F).

The overall proportion of DMIs captured from known DMIs revealed that BioGrid achieved the highest percentage (31%) of DMIs from the known human DMIs dataset (1236 known human DMIs). Following closely, HPRD captured 26% of the DMIs. In comparison, all other datasets captured a lower proportion of DMIs than these two (Figure 1G). Similarly, the overall proportion of DDIs captured from the total known DDIs demonstrated that HIPPIE secured the highest percentage (∼48%) of DDIs from the total known DDIs dataset (5589 DDIs). BioGrid followed with 45% of DDIs captured, HPRD with 37% DDIs, and IntAct with 23% DDIs. Like the DMI analysis, all other datasets exhibited a lower proportion of DDIs compared to these datasets (Figure 1G).

Upon close examination of our analysis, less than 1% of DMIs and less than 3% of DDIs can be explained by the known DMIs and DDIs. Furthermore, a minimal number of PPIs were discovered to be both DMIs and DDIs (∼3%) (Figure 1H). To evaluate the significance of real DMIs in each dataset in relation to other datasets, a chi‐square pairwise test was conducted. All datasets showed significant differences (*p* < 0.001) when compared to each other except CoFrac‐12 compared to CoFrac‐15.

### 3.2 Exploring the ability of binary and co‐complex interactions to detect DMIs and DDIs

As both the BioPlex2.0 and HI‐II‐14 datasets exhibited substantial enrichment (∼106×, 81×), suggesting that both Y2H and AP‐MS screens effectively captured DMIs, we opted to delve deeper into determining which method, Y2H or AP‐MS, performed better in capturing DMIs. We conducted a binary versus co‐complex PPI analysis, separating binary PPIs (Y2H) and co‐complex PPIs (AP‐MS and CoFrac‐MS) from BioGrid, IntAct, and HIPPIE databases to assess enrichment. Both binary and co‐complex PPIs displayed significant DMI enrichment when compared to random protein pairs. While all methods captured a notable number of known DMIs, the Y2H method exhibited higher DMI enrichment (*p*‐value <0.001) compared to other methods. We also examined which method, among AP‐MS, Y2H, and CoFrac‐MS, excelled in capturing DDIs. Once again, the Y2H method demonstrated higher enrichment than the other methods (*p* < 0.001). In summary, Y2H screens showed superior enrichment compared to other methods in capturing both DMIs and DDIs (Table 2).

**TABLE 2:** Enrichment of DMIs and DDIs in high‐throughput PPIs.

Dataset	Method	PPIs^a^	potDMIs^b^	DMIs^c^	DMI enrichment^d^ (4 s.f.)	potDDIs^e^	DDIs^f^	DDI enrichment^g^ (4 s.f.)
BioGrid	AP‐MS	95,034	427	80	23.82**	1871	540	25.39**
	Two‐hybrid	44,569	603	98	56.06**	2723	672	75.36**
	CoFrac‐MS	37,532	245	22	48.03**	1979	392	18.15**
IntAct	AP‐MS	17.926	223	27	16.47**	1757	184	8.83**
	Two‐hybrid	17,533	378	35	28.41**	1980	220	22.06**
HIPPIE	AP‐MS	13,675	435	94	31.28**	1532	294	43.5**
	Two‐hybrid	95,403	778	121	49.94**	3548	792	74.95**
	CoFrac‐MS	281	10	3	27.52**	43	20	29.07**

Abbreviations: CoFrac‐MS, Co‐Fraction‐coupled Mass Spectrometry; DDI, domain–domain interaction; DMI, Domain Motif Interactions; HIPPIE, Human Integrated Protein–Protein Interaction rEference; PPI, Protein–protein interaction.

^a^ Number of nonredundant, reviewed, and symmetrical PPIs.

^b^ All potential DMIs.

^c^ Known DMIs captured by different datasets.

^d^ Enrichment of known DMIs.

^e^ All potential DDIs.

^f^ Known DDIs captured by different datasets.

^g^ Enrichment of DDIs.

**p‐value <0.001.

### 3.3 The enrichment experienced a decline with the introduction of noise into the DMI network

**FIGURE 2:**
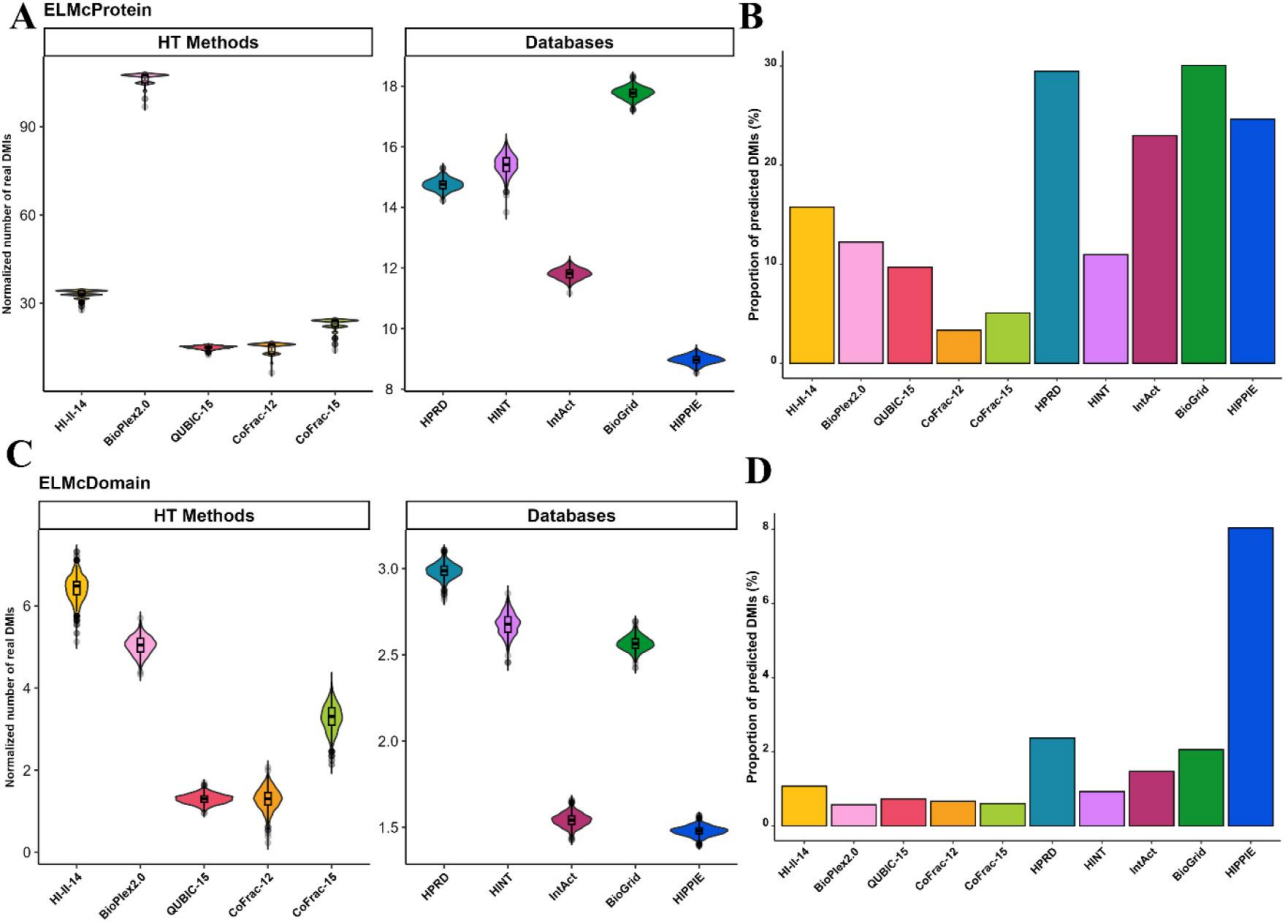
Predicting DMIs utilizing ELMc‐Protein and ELMc‐Domain strategies. (A) Normalized number of DMIs captured over 1000 randomizations using the ELMc‐Protein strategy. (B) Total proportion of DMIs predicted from potential DMIs using the ELMc‐Protein strategy. Potential DMIs represent the overall proportion of those DMIs that could theoretically be identified based on the proteins in the PPIs. (C) Normalized number of DMIs captured over 1000 randomizations using the ELMc‐Domain strategy. (D) Total proportion of predicted DMIs captured from potential DMIs using the ELMc‐Domain strategy. DMI, Domain Motif Interactions; ELM, Eukaryotic Linear Motif; ELMc‐Domain, Protein Interactions via ELM Class and Pfam Domains; ELMc‐Protein, Known ELM Instances Interacting Proteins.

We employed noisier DMI predictions to augment the identification of real DMIs as only a small fraction of known DMIs was captured by high‐throughput datasets. The objective was to assess whether the general pattern of enrichment remained consistent. Initially, the ELMc‐Protein strategy was implemented, wherein known SLiMs were mapped onto their respective protein partners through ELM classes. While all datasets maintained significant enrichment (*p*‐value <0.001) over random expectation, the overall enrichment scores decreased for most datasets compared to the ELMi‐Protein strategy (Figure 2A). Subsequently, the ELMc‐Domain strategy, incorporating domain information, was employed. Despite a further decline in enrichment scores, the overall trend of this strategy remained consistent with that of ELMc‐Protein (Figure 2C, Table 3). The total proportion of predicted DMIs captured from potential DMIs, representing the theoretical identifiability of DMIs given the proteins in the PPI datasets, was then calculated. Using the ELMc‐Protein strategy, BioGrid, HPRD, and HIPPIE databases predicted the highest proportion of DMIs from potential DMIs. Among high‐throughput screens, HI‐II‐14 identified the highest proportion of DMIs, followed by BioPlex2.0, QUBIC‐15, CoFrac‐15, and CoFrac‐12 (Figure 2B). Conversely, only a small fraction (1%–8%) of DMIs was predicted from potential DMIs through the ELMc‐Domain strategy, with HIPPIE having the highest proportion of predicted DMIs from potential DMIs (Figure 2D). Despite the increase in the number of DMIs with the elevation of noise in DMI prediction quality, the high enrichment of these datasets suggests that the additional DMIs are likely to be authentic (Table 3).

**TABLE 3:** Noisier DMI prediction strategies.

DMI prediction strategy	Dataset	Method	potDMIs^a^	DMIs^b^	Enrichment^c^ (4 s.f.)	FDR^d^
ELMc‐protein	HI‐II‐14	Y2H	165	26	34.26**	0.0219
	BioPlex2.0	AP‐MS	327	40	107.5**	0.0093
	QUBIC‐15	AP‐MS	516	50	15.99**	0.062
	CoFrac‐12	CoFrac‐MS	150	5	16.03**	0.0624
	CoFrac‐15	CoFrac‐MS	236	12	24.14**	0.0414
	HPRD	All	1510	445	15.75**	0.063
	HINT	All	1330	146	16.42**	0.060
	IntAct	All	1575	362	12.8**	0.078
	BioGrid	All	1617	486	18.78**	0.053
	HIPPIE	All	1652	407	9.97**	0.1003
ELMc‐domain	HI‐II‐14	Y2H	6626	71	7.4**	0.134
	BioPlex2.0	AP‐MS	19,404	110	6.03**	0.1657
	QUBIC‐15	AP‐MS	22,979	166	2.29**	0.4353
	CoFrac‐12	CoFrac‐MS	4526	30	2.29**	0.4354
	CoFrac‐15	CoFrac‐MS	6708	40	4.26**	0.2344
	HPRD	All	83,239	1971	3.98**	0.250
	HINT	All	84,954	784	3.67**	0.272
	IntAct	All	97,391	1433	2.54**	0.3934
	BioGrid	All	95,091	1957	3.56**	0.280
	HIPPIE	All	110,042	8843	2.47**	0.403

Abbreviations: DMI, Domain Motif Interactions; FDR, False Discovery Rate; HINT, High‐quality INTeractome; HIPPIE, Human Integrated Protein–Protein Interaction rEference; HPRD, Human Protein Reference Database; Y2H, Yeast two‐hybrid.

^a^ Potential DMIs count.

^b^ DMIs predicted in the PPIs.

^c^ Enrichment of DMIs.

^d^ FDR of the predictions.

**p‐value <0.001.

### 3.4 High‐quality PPIs can be a good source of capturing DMIs

**FIGURE 3:**
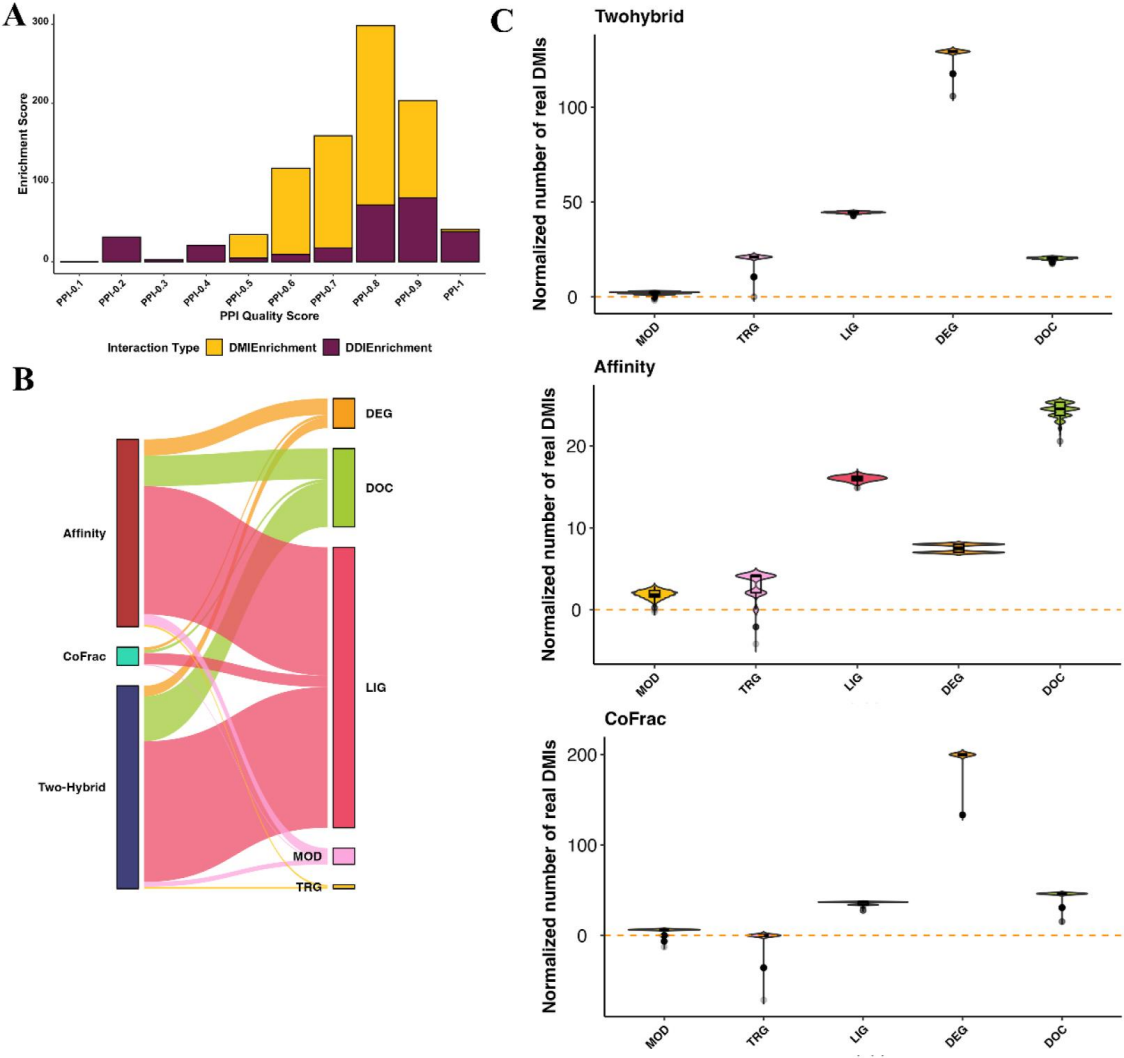
Impact of PPI quality and ELM types. (A) Impact of PPI quality on DMI and DDI enrichment: The *X*‐axis depicts the confidence scores of various PPI subsets sourced from the HIPPIE database (ranging from 0.1 to 1), while the *Y*‐axis represents the enrichment score of PPIs within distinct subsets based on their confidence scores. (B) Influence of ELM types on DMI enrichment in interactions from three high‐throughput methods (AP‐MS, Y2H, and CoFrac‐MS) obtained from PPI databases. (C) The normalized count of real DMIs captured by different ELM classes. CoFrac‐MS, Co‐Fraction‐coupled Mass Spectrometry; DDI, domain–domain interaction; DMI, Domain Motif Interactions; ELM, Eukaryotic Linear Motif; HIPPIE, Human Integrated Protein–Protein Interaction rEference; PPI, Protein–protein interaction; Y2H, Yeast two‐hybrid.

We delved deeper into understanding how the quality of PPIs could influence DMI enrichment. Specifically, we focused on the HIPPIE dataset and assessed the enrichment for PPIs with varying confidence scores (ranging from 0 to 1, where 1 represents highly confident PPIs). Our aim was to explore how the confidence score impacted overall DMI enrichment. The quality of PPIs indeed had an influence on enrichment. PPIs with higher confidence scores (0.6–0.9) exhibited greater enrichment in terms of capturing known DMIs (Figure 3A). We extended this analysis to evaluate the impact of PPI quality on DDI enrichment. However, there was not a straightforward correlation between PPI confidence and DDI enrichment. Once again, the optimal confidence score for DDI prediction fell between 0.8 and 0.9 (Figure 3A).

### 3.5 Different ELM types can have an impact on DMI enrichment

The ELM database encompasses six distinct ELM types, namely cleavage (CLV), degron (DEG), docking (DOC), ligand (LIG), post‐translational modification sites (MOD), and targeting (TRG). To investigate whether specific ELM types influenced the effectiveness of PPI detection techniques in capturing DMIs, we examined the enrichment for individual ELM types using the ELMc‐Protein strategy. This analysis involved amalgamating high‐throughput PPI data from BioGrid, HIPPIE, and IntAct databases. Concerning predicted DMIs, all methods exhibited a higher capture of LIG‐mediated interactions compared to other ELM classes, while no CLV‐mediated interactions were identified (Figure 3B). Real DMIs, observed over 1000 randomizations, indicated a significant presence of DEG‐mediated DMIs but did not capture a substantial amount of CLV (Figure 3C). Notably, AP‐MS PPIs captured more LIG & DOC‐mediated real DMIs, while Two‐hybrid and CoFrac methods captured more DEG‐mediated DMIs. In summary, this analysis suggests that different ELM types can have varying impacts on the detection of DMIs.

## 4 DISCUSSION

Various methods have emerged to explore DMI, but most of them focus on specific domain families (PDZ, SH2, SH3, and WW), leaving gaps in our understanding.^3^ Contemporary studies aim to identify SLiMs alongside their binding partners in the human proteome.^41^, ^42^ While low‐throughput studies have contributed to DMI knowledge, the efficiency of high‐throughput methods in capturing different interactions, including DMIs and DDIs, remains unexplored. To address this, we compared PPIs from different methods and databases, assessing their ability to capture DMIs and DDIs. A challenge in PPI analysis lies in inconsistent protein identifiers across datasets.^43^ To ensure uniformity, we mapped datasets lacking Uniprot IDs (*e.g.,* CoFrac‐12, CoFrac‐15, HI‐II‐14, HIPPIE, BioGrid, and HPRD) to their respective Uniprot IDs to facilitate reliability and mitigate redundancy issues. The resulting number of interactions, pre‐ and post‐mapping, instilled confidence in the datasets for subsequent analysis. Evaluation of high‐throughput screens (e.g., HI‐II‐14, BioPlex2.0, CoFrac‐12, CoFrac‐15, and QUBIC‐15) revealed significant enrichment in capturing DMIs compared to random protein pairs. Despite capturing less than 1% of known DMIs, the substantial enrichment fold suggests the authenticity of the captured interactions. High throughput methods, specifically Y2H and AP‐MS, demonstrated comparable or superior performance to curated PPI databases in DMIs enrichment.

Assessment of DDI capture by different datasets showed significant enrichment across the board, with BioPlex2.0 and HI‐II‐14 exhibiting higher enrichment than CoFrac datasets. However, only a small fraction of PPIs could be attributed to known DDIs, emphasizing the limited understanding of these interactions. Known DMI data captured by diverse datasets accounted for less than 1% of PPIs, aligning with literature indicating the scarcity of known DMIs.^28^ Analysis of known DDI data from 3DID revealed less than 3% of coverage, implying potential gaps in high‐throughput methods in capturing these interactions. Schuster‐Bockler and Bateman suggest that the existing DDI data within iPfam can account for only a fraction, specifically 4%–19%, of protein interactions in *Homo sapiens*.^44^ This underscores the worry that a significant portion of DMIs/DDIs remains undiscovered. The quantity of recognized DMIs and DDIs in PPIs, along with their enrichment, underscores the significance of protein composition in identifying these interactions. HIPPIE exhibited a greater proportion of known DMIs compared to other databases. Conversely, high‐throughput methods demonstrated a lower proportion of known DMIs when contrasted with curated databases.

The two distinct methods for mapping PPIs involve binary interactions, where two proteins have direct physical contact and co‐complex interactions, which usually require additional proteins to form multimeric complexes. These complexes may encompass both direct and indirect interactions among various proteins. While Y2H is renowned for identifying binary interactions, AP‐MS and CoFrac‐MS are employed to detect co‐complex interactions.^4^ Given the higher enrichment fold observed in both BioPlex2.0 and HI‐II‐14 datasets (∼106×, 81×), we delved deeper into determining which method, binary or co‐complex, excelled in identifying DMIs and DDIs. We extracted binary PPIs (Y2H) and co‐complex PPIs (AP‐MS and CoFrac‐MS) from three renowned databases (BioGrid, IntAct, and HIPPIE) for the enrichment evaluation. In prior studies,^45^, ^46^ binary approaches have been utilized to identify DMIs, such as the identification of SUMO interacting motifs that interact with SUMO1 and SUMO2 proteins.^45^, ^46^ However, there are no specific studies employing co‐complex approaches to discover DMIs. Our analysis revealed significant DMI enrichment in all datasets compared to random protein pairs, with no clear winner between Y2H and CoFrac data. Y2H and CoFrac data notably captured degron motif‐mediated DMIs, while AP‐MS captured more conventional ligand‐mediated DMIs. ELM‐type analysis indicated that CLV and MOD were generally not effective at capturing DMIs. The rationale behind other ELM types capturing significant DMIs, while CLV and MOD could not, might be related to motif complexity or their low complexity nature, and involvement in PTMs. Overall, the analysis revealed that MOD and CLV types were generally not effective at capturing DMIs. In future studies, it would be intriguing to conduct a comparative analysis, evaluating the efficacy of other different methods, including BioID Mass Spectrometry^47^ and Phage Display,^48^ in capturing DMIs and DDIs.

As evident, large PPI databases contain numerous interactions, but only a fraction is known to be mediated by DMIs. Examining the enrichment patterns of these databases revealed a limitation in enrichment due to the low number of known DMIs, indicating a substantial reservoir of undiscovered DMIs. To investigate whether introducing noise through DMI prediction methods could unveil additional DMIs with significant enrichment, we employed various strategies available in SLiMEnrich. In recent years, PPI data has been combined with computational tools to discover new DMIs for recognized domains such as PDZ, WW, SH3, and SH3 domains (e.g., 49, 50, 51, 52). In our approach, we integrated PPI data with motif and domain information available in the ELM database for DMI predictions. The introduction of noise in the DMI network, using motif and/or Pfam domain, increased the number of predicted DMIs, albeit with a decline in the overall enrichment score for datasets. Generally, the ranking and trend of dataset enrichments remained consistent when employing ELMc‐Protein or ELMc‐Domain strategies, aligning with previous studies that noise in the DMIs diminishes enrichment scores.^39^ ^,53^ The predicted DMIs from the ELMc‐Protein strategy exhibited a notably low FDR for high‐throughput datasets, suggesting their potential authenticity. Conversely, the estimated FDR for individual DMI predictions was relatively high (0.1–0.4) for the ELMc‐Domain strategy, emphasizing the need for caution in interpreting large‐scale predictions of this nature. Overall, diverse DMI predictions indicated substantial enrichment in these databases. Motif predictions from different tools, such as SLiMProb, incorporating conservation masking, could potentially yield less noisy predictions. Future investigations could explore the impact of such predictions on the quality of DMI predictions. Assessing whether PPI quality contributed to enrichment, we evaluated enrichment in PPIs from the HIPPIE database based on their confidence score. Higher‐confidence PPIs showed promise in capturing DMIs, suggesting that curated and high‐quality PPIs might serve as a valuable source for capturing DMIs. However, examining the DDI enrichment trend revealed no consistent correlation with confidence scores, indicating that confidence alone may not equate to data quality for DDI predictions.

## 5 CONCLUSION

The surge in high‐throughput experimental methods has led to the generation of extensive PPI datasets. Given the error‐prone nature of these methods, there is a growing need for new approaches to evaluate the PPIs as potential sources of various interaction types, such as DMI or DDI. In this study, we scrutinized PPIs derived from various publicly available resources to gauge their efficacy in capturing DMIs and DDIs. Our findings revealed significant enrichment across all databases, with both Y2H and AP‐MS showing promise in capturing DMIs and DDIs. This positions them as potential methods of choice when exploring interactions involving domains and motifs.

## AUTHOR CONTRIBUTIONS

Sobia Idrees designed and performed the experiments, analyzed the data, contributed analysis tools, prepared figures and/or tables, authored or reviewed drafts of the paper, and approved the final draft. Keshav Raj Paudel authored or reviewed drafts of the paper and approved the final draft.

## CONFLICT OF INTEREST STATEMENT

The authors declare that they have no conflict of interest.

## ETHICS STATEMENT

Not applicable.

## Data Availability

This article contains excerpts from Idrees' thesis published in 2020 (Idrees 2020).
